# Book Review

**Published:** 1913-06

**Authors:** 


**Flatulence and Shock.** By F. G. Crookshank, M.D. Lond., M.R.C.P. Pp. 47. London: H. K. Lewis. 1912. Price 2s.—These two essays are of much interest, and should be carefully perused. Common things such as "windy spasms" demand more consideration than they often receive. Among many other causes the author emphasises the importance of gas secretion from the walls of the stomach and bowels, to which air gulping is added to overcome the spasm that prevents eructation. The author concludes his essay with a practical suggestion likely to be of service not unfrequently: "Should any of you, from overwork, overmuch tea, overmuch tobacco, and what not, get a spell of neurotic flatulence and eructation with air gulping, nothing will give you so much relief as a bottle of the very best Crème de Menthe, sipped slowly and steadily until you are better." The author emphasises the importance of recognition of "delayed shock," when death may occur in an aged person a week or two after a slight fall that has involved fracture of the femur. Death is clearly the result of the accident, even although the obvious lesion appears to be of little danger to life.

**Treatment after Operation.** By William Turner, M.S., F.R.C.S., and E. Rock Carling, B.S., F.R.C.S. Pp. x, 247. London: University of London Press. 1912. 10s. 6d. net.—This small and most useful book describes in a simple way all the essentials of treatment after surgical operations. The opening chapters deal with the sick room, anæsthesia, asepsis, hemorrhage, and operations on various tissues; and the rest of the book is concerned with the operations of regional surgery, ending with a chapter on the eye by L. V. Cargill. No attempt is made to review or discuss all possible methods of treatment, but only those which are in practical use by the authors are described. It is not, therefore, an exposition of anything new, but rather a helpful guide for students or house surgeons.

**Gonococcal Infections.** By Major C. E. Pollock, R.A.M.C. and Major L. W. Harrison, R.A.M.C. Pp. xxi, 222. London:

Oxford Medical Publications. 1912.—The account given of gonorrhœa and its complications and of the other gonococcal infections given in this book is an excellent one. It is short, succinct and practical, and will be sufficient for the great majority of practitioners. Pathology, vaccine and serum treatment, and complications, both in the male and female, receive due attention. The most modern methods of treatment are described with sufficient detail to enable the practitioner to adopt them, and guidance is given in the selection of methods, a guidance based upon the writers' large experience. We can cordially recommend the book.

**A Text-Book of Pathology.** By J. GEORGE ADAMI, M.A., M.D., F.R.S., and JOHN MCCRAE, M.D., M.R.C.P. Pp. x, 17–759. London: Macmillan & Co. Ltd. 1912. 25s. net.—The book is admirably adapted to the requirements of the medical student, for whom it has been written. It is divided into two parts, dealing with general and special and systemic pathology. The authors have endeavoured to make clear and intelligible what is known concerning the deeper meaning of morbid states, and in so doing have certainly succeeded in giving a remarkably clear account of many abstruse subjects. The book is well illustrated by numerous engravings in the text, and also by eleven coloured plates.

**Sleeping Sickness.** By Captain F. P. MACKIE, I.M.S. Pp. 38. Calcutta: Superintendent Government Printing. 1912.—The pamphlet gives a summary of the work done by the Sleeping Sickness Commission (1908–1910), of which Capt. Mackie was a member. The Commission concerned itself chiefly with the question of sanitation and transmission of sleeping sickness by biting insects. Treatment, even if experimentally a success, is practically out of the question under the conditions prevailing in Africa. The reviewer ventures to consider that one of the most important results of this expedition is the observation that Glossina Palpalis fed on natives who have been treated with arsenic compounds and other drugs is capable of transmitting sleeping sickness (p. 27). Recurrent trypanosomes, after arsenic treatment, are often very virulent, and this fact emphasises the great danger which is involved in the treatment of thousands of natives with drugs which only cause a temporary disappearance of the trypanosomes. Such a mistake has been made by Koch, and is greatly to be regretted.

**Microbes and Toxins.** By D. ETIENNE BURNET. With a Preface by ELIE METCHNIKOFF. Translated by Dr. CHARLES BROQUET and W. M. SCOTT, M.D. Pp. xvi, 316. London: Wm. Heinemann. 1912. Price 5s.—**Clinical Immunity and Sero-Diagnosis.** By A. WOLFF-EISNER, M.D. Translated by

Ray W. Matson, M.D. Pp. xiv., 184. London: Baillière, Tindall & Cox. 1911. Price 7s. 6d.—Both of these small books may be read with advantage by the student of bacteriology. Of the two, however, Dr. Burnet's book will be found to be more suitable for those not engaged in active bacteriological research. While it is not by any means a text-book on the subject, as its name might imply, it nevertheless deals with the broad underlying facts of bacteriology, at least so far as we know them. It begins with a rapid bird's-eye view of organisms in general, and their place in Nature, and gradually leads on to the great problems of immunity. As one would expect, the "phagocyte" dominates this part of the work, but the writer is broad enough to state fairly the humoralistic theory. Vaccines and sero-therapy are also discussed. It has been exceptionally well translated by Drs. Scott and Broquet, and contains much that is stimulating and suggestive. It can be read easily by the practitioner whose knowledge of the subject is limited, and should certainly be read by every student. The second book, while essentially one for more advanced workers in bacteriology, is too full of "Wolff–Eisner." We should not like to place it in the hands of a "tyro," if we desire him to have a fair idea of the subject. Not that it is of little interest, for it is full of suggestions, but it is too one-sided. The author is possessed of the new idea of anaphylaxis, and he attempts to make the observed facts of infection and immunity fit this idea. There is much in the book that is open to discussion, for much of it is entirely without proof. For the advanced researcher it is to be commended. For others, however, the first-named book will be of greater value.

**Bacteria.** By Dr. Max Schottelius. Translated by Staff-Surgeon Herbert Geoghegan, R.N. Second Edition. Pp. xii, 324. Ten plates. London: Oxford Medical Publications. 1912. 8s. 6d. net.—This is a popular, discursive, attractively-illustrated sketch of bacteriology for the general public. It begins with a survey of the position and use of bacteria in Nature; then describes bacteriological research methods, infectious diseases in general, and the prevention of infection by means of disinfectants, immunisation and other means. Next special diseases are taken as examples, and the work finishes with a chapter on protozoa. The advantages of public health administration and a knowledge of hygiene are indicated in the descriptions of small-pox and dysentery. Digressions are common. For instance, "Teetotal fanatics will not reach heaven" (page 7), and the opinion that teachers should rank "above the confectioners with their gay little tarts" (page 10); but surely the emphasis of the specialisation of bacteria, particularly the parasitic species dangerous to man, would have been more in place. The spoiling of fine furs (page 132)

is a serious matter, but the use of formalin in foods is a more serious objection, though this has escaped the notice of the author. These are, however, details. The book is easily read, and cannot obviously give very detailed information.

**Puerperal Infection.** By ARNOLD W. LEA, M.D., B.S., F.R.C.S. Pp. xlvi, 384. London: Oxford Medical Publications. 1910. Price 25s.—We regard this work as one of the most important monographs on the subject that has yet appeared, giving as it does the completest information as to the history, causation, bacteriology, pathology, symptoms and treatment of this affection. There are several excellent points in the arrangement of the work worth noting. The index is most complete and thorough, the plates are numerous and clear, showing a high degree of artistic merit, while the arrangement of the bibliographical references is most careful, a complete list being given at the end of each chapter. No reference of importance is omitted, and the large number quoted makes the book invaluable as a work of reference. The author's style is easy and pleasant, and the print large and clear. Space will not permit of such a full consideration of the contents as is demanded by their importance, but we would commend specially for study the statistical tables given, and the author's remarks thereon. He produces good evidence to show that the seasonal incidence of puerperal infection is a fact the explanation of which is comparatively simple, but none the less of first-class importance, and that while air-borne infection is comparatively uncommon, yet climatic conditions have a most important influence on the cure incidence of the disease. He also rightly lays stress on the frequency of the occurrence of infection in cases of toxæmia of other than septic origin. This fact, well known as it is to obstetricians, cannot be too strongly emphasised. His account of the bacteriology is most illuminating, and his insistence on the frequency of mixed infections and the infrequency of pure ones a point that requires general emphasis. He does not consider that the work so far presented on serum treatment is sufficiently definite in its results to allow of this method being regarded as a specific means of dealing with the affection, but his presentation both of this method and the treatment by vaccines is most valuable and complete. He gives a lucid and full account of the different operative procedures required, and lays down one sound rule which may render the decision as to whether hysterectomy is called for or not less difficult than it usually is. His suggestion is that the puerperal uterus should be looked upon in the same way as the non-puerperal, and that just as we should advise and practise hysterectomy in the case of an infected fibroid, so we should adopt a similar course in the case of a limited local infection of the puerperal uterus. We can only say that any one who wishes to consult a work of reference on the subject will find no clearer or fuller information on any point he may wish to ascertain.

**Transactions of the American Association of Obstetricians and Gynæcologists.** Vols. XIX and XX. New York. 1906–7.—These volumes contain many interesting records of cases and discussions. Vol. XIX contains an interesting case and discussion of Porro Cæsarean section during pregnancy in a patient in whom irrigation of the membranes with prolapse of cord, but without labour pains, occurred in a patient suffering from a fibroid in the lower segment of the uterus, filling the pelvis completely. Both mother and child recovered. Several similar cases were narrated in the following discussion, which contained several references to myomectomies performed during pregnancy. Papers on "Chronic Dyspepsia resulting from Pelvic and Abdominal Disease" (Herman Hayd), "The Diagnosis and Treatment of Accidental Hemorrhage" (Adam H. Wright), "Anterior Vaginal Celiotomy" (Saml. Bandler), "Some Observations and Experiences Respecting the Symptoms and Treatment of Cases of Atresia of the Vagina" (Augustus Clarke), will all repay perusal. Amongst other papers in Vol. XX, one by Raleigh R. Huggins on "The Toxæmias of Pregnancy" deserves notice The author, among other acute observations, notes the liability of sufferers from pregnancy toxæmias to the development of septic infection in the puerperium, and also relates cases which throw some light on the curious cases of jaundice met with at times as a complication of the puerperium. Hayd relates the removal, with success, of a lithopedion from a woman aged 67. James W. West also in a paper on "When shall we Perform Myomectomy and when Hysterectomy in Uterine Fibromyomata?" gives the pros and cons of the merits of the respective operations with great clearness, and also lays down distinctly the rules which should govern the treatment by operation generally. James E. Sadlier on "Consistency in Aseptic Surgical Technique" makes some rather pregnant remarks on technique and consistency which will be found valuable. Edward J. Ill in a paper on "The Conservative Medical Treatment of Salpingitis" presents a valuable corrective to those who consider operation the one and only treatment for inflammatory affection of the uterine appendages. He gives one or two valuable suggestions as to treatment, and also as to cases in which reinfection sometimes periodic in character takes place. The paper is one well worthy of study.

**Gynæcological Therapeutics.** By S. Jervois Aarons, M.D., M.R.C.P. Pp. xiv. 178. London: Baillière, Tindall & Cox. 1910. 5s. net.—This little volume is written for the use of the general practitioner as a handy reference book, containing definite and concise directions for the treatment of those gynæcological affections which fall within his province, and as such should be warmly welcomed by him. No account of pathology or diagnosis is attempted, the work dealing simply with the question of treatment. The result is that this book contains within small compass a collection of prescriptions, with practical hints on their use and the necessary instructions to patients, which must be most useful to anyone embarking on general practice with only the amount of knowledge and experience in this department acquired in the course of the ordinary curriculum. Particularly good are the chapters on affections of the external genitals and on constipation. The former furnishes the practitioner with a wide choice of remedies to which he may turn in those perplexing cases of pruritus, eczema vulvæ, etc., which at times tax his ingenuity in therapeutics, while the latter contains most helpful advice on the rational treatment of cases of simple constipation. Some useful prescriptions will also be found on those pages dealing with dysmenorrhœa, but no mention is made of guaiacum in the treatment of this complaint, although some cases are very markedly relieved by this drug. The fact that this little book contains at any rate a summary of all the medical side of gynæcological treatment, serves to show clearly how essentially surgical this department of our art has become.

